# Pathognomonic Sign of Fracture of the Neck of the Femur

**Published:** 1880-12

**Authors:** 


### PATHOGNOMONIC SIGN OF FRACTURE OF THE NECK OF THE FEMUR.

DR. BEZZI draws attention, in *Lo Spallanzani*, Nos. 1 and 2, 1880, to a sign which is pathognomonic of fracture of the neck of the femur, but which is not generally known. In examining the space between the trochanter and the crista ilii, it will be found that while, on the sound side, the muscles occupying this region (the tensor vaginæ femoris and the gluteus medius) are tense, and offer to the hand a considerable feeling of resistance, they present on the affected side a deep, well marked depression, a flaccidity and diminution of tension, from displacement upward of their points of insertion.

